# Levels of plasma PAD4 and citrullinated vimentin in relation to clinical severity of periodontitis and their exploratory discriminatory performance

**DOI:** 10.3389/fcimb.2026.1949560

**Published:** 2026-10-12

**Authors:** Weiwen Hu, Zhijia Xu, Xinyu Li, Xiaoyu Sun, Jiajie Tu

**Affiliations:** 1 Institute of Clinical Pharmacology, School of Pharmacy, Anhui Medical University; Key Laboratory of Anti-inflammatory and Immune Medicine (Anhui Medical University), Ministry of Education, Hefei, China; 2 College & Hospital of Stomatology, Anhui Medical University, Anhui Province Key Laboratory of Oral Diseases Research, Hefei, China

**Keywords:** biomarker, citrullinated vimentin, peptidylarginine deiminase 4, periodontitis, protein citrullination

## Abstract

**Objective:**

To compare plasma peptidylarginine deiminase 4 (PAD4) and citrullinated vimentin (cit-VIM) concentrations between patients with periodontitis and screened controls and to explore their associations with periodontal clinical parameters and their discriminatory performance within the present sample.

**Methods:**

A cross-sectional case-control study was conducted. A total of 30 periodontally healthy subjects and 60 patients with periodontitis were enrolled. Peripheral venous blood samples were collected for plasma isolation, and the concentrations of PAD4 and cit-VIM were determined by enzyme-linked immunosorbent assay (ELISA). Clinical parameters including maximum probing depth (PDmax), plaque index (PLI), calculus index (CI), maximum tooth mobility, maximum relative radiographic bone loss (RBLmax), and periodontitis stage were recorded. The levels of the two biomarkers were compared between the two groups. Spearman rank correlation analysis was used to evaluate associations between plasma biomarkers and periodontal variables, with Benjamini–Hochberg correction for multiple testing. Binary logistic regression was performed to evaluate the associations of PAD4 and cit-VIM with periodontal status after adjustment for age, sex, and smoking. Receiver operating characteristic (ROC) curves, DeLong comparisons, Youden-index-derived cut-offs, and bootstrap internal validation were used to assess exploratory discriminatory performance.

**Results:**

Plasma PAD4 and cit-VIM concentrations were significantly higher in the periodontitis group than in the healthy control group (both P<0.001). PAD4 and cit-VIM were positively correlated in patients with periodontitis (r_s_=0.459, P<0.001). After Benjamini–Hochberg correction, cit-VIM remained associated with PDmax and PLI, whereas PAD4 remained associated with PDmax, PLI, CI, RBLmax, and periodontitis stage. In the adjusted logistic regression model, PAD4 (OR = 2.90, 95% CI: 1.21–6.96, P = 0.017) and cit-VIM (OR = 12.70, 95% CI: 3.43–46.96, P<0.001) remained associated with periodontitis. The areas under the ROC curves (AUCs) for PAD4, cit-VIM, and their combined model were 0.803, 0.931, and 0.946, respectively. The combined model outperformed PAD4 alone (P = 0.001) but not cit-VIM alone (P = 0.385), and its bootstrap optimism-corrected AUC was 0.941.

**Conclusion:**

Plasma PAD4 and cit-VIM were associated with periodontal status and demonstrated exploratory discriminatory ability within the present sample. Their potential complementary value requires validation in larger independent cohorts.

## 1 Introduction

Periodontitis is a chronic destructive disease driven by the imbalance between the dental plaque biofilm and host immune-inflammatory responses (Caton et al., 2018; Hajishengallis and Lamont, 2021; Lin et al., 2021; Papapanou et al., 2018), characterized mainly by loss of periodontal attachment, periodontal pocket formation and alveolar bone resorption. In the 2017 World Workshop classification, periodontitis stage reflects disease severity and treatment complexity (Papapanou et al., 2018; Tonetti et al., 2018). In the present study, stage was included as a clinical indicator of accumulated periodontal destruction to explore its association with plasma biomarker levels. Clinical diagnosis primarily relies on probing depth, clinical attachment level, and radiographic bone loss. Plasma biomarkers are not substitutes for these established diagnostic criteria but may provide complementary information on inflammatory and tissue-destructive processes. However, plasma biomarker concentrations may also reflect systemic inflammatory burden and therefore may have limited specificity for periodontitis (Andreu et al., 2021; Isola, 2021; Rakic et al., 2025).

Protein citrullination is a post-translational modification catalyzed by peptidylarginine deiminases (PADs), which convert arginine residues in proteins into citrulline, thereby altering protein charge, conformation and immunogenicity (Mondal and Thompson, 2019). Peptidylarginine deiminase 4 (PAD4) is predominantly expressed in myeloid cells such as neutrophils and participates in histone citrullination and neutrophil extracellular trap (NET) formation (Chen et al., 2022; Liu et al., 2021; Wang et al., 2021). Engström et al. reported increased staining of citrullinated proteins and higher PAD2 and PAD4 protein expression in the gingival connective tissue of patients with periodontitis, whereas corresponding differences were not observed in the gingival epithelium (Engström et al., 2018). The same study also reported increased PADI2 and PADI4 mRNA levels in whole gingival biopsies, and these changes were not correlated with the detected presence of *P. gingivalis* or *A. actinomycetemcomitans* leukotoxin (Engström et al., 2018). Harvey et al. detected PAD2, PAD4, and citrullinated proteins in human gingival tissue and reported that their tissue protein expression was associated with the severity of local inflammation; however, PADI2 and PADI4 mRNA were detectable in both inflamed and non-inflamed gingival tissues (Harvey et al., 2013).Nesse et al. demonstrated increased global citrullinated-protein staining in the connective-tissue stroma of periodontitis-affected samples but did not specifically assess PAD expression, citrullinated vimentin, or circulating biomarkers (Nesse et al., 2012).A recent systematic review similarly concluded that current periodontal evidence primarily concerns local PAD expression, PAD activity, and global protein citrullination, whereas direct evidence regarding circulating citrullinated vimentin (cit-VIM) remains limited (Bonilla et al., 2024). In addition to host PAD4, *Porphyromonas gingivalis* expresses a bacterial peptidylarginine deiminase (PPAD) with substrate specificity distinct from that of mammalian PADs (Abdullah et al., 2013). The potential contribution of host PADs and bacterial PPAD to periodontal protein citrullination has been summarized in a recent systematic review (Bonilla et al., 2024). Host PAD4 and bacterial PPAD are distinct enzymes and should not be interpreted as interchangeable sources of protein citrullination. Vimentin is an intermediate filament protein susceptible to citrullination. Experimental evidence indicates that extracellular cit-VIM can promote osteoclastogenesis and alveolar bone resorption in a mouse model of periodontitis (Shindo et al., 2023). This biological relationship provides a rationale for evaluating plasma PAD4 and cit-VIM together. However, plasma PAD4 concentration does not represent PAD4 enzymatic activity, and concurrent elevations of PAD4 and cit-VIM cannot establish direct PAD4-mediated vimentin citrullination. Their plasma concentrations may also reflect systemic inflammatory burden rather than a periodontitis-specific molecular process.

Recent evidence indicates that combinations of inflammatory biomarkers may improve discrimination compared with individual markers; however, their clinical translation remains limited by assay heterogeneity, insufficient validation, and limited specificity for periodontal inflammation (Litsou, 2026).

This exploratory study hypothesized that plasma PAD4 and cit-VIM concentrations would differ between periodontally healthy individuals and patients with periodontitis and would be associated with clinical and radiographic measures of periodontal disease burden. The primary objective was to compare plasma PAD4 and cit-VIM concentrations between the two groups and to evaluate their mutual association and their associations with maximum probing depth (PDmax), plaque index (PLI), calculus index (CI), maximum tooth mobility, maximum relative radiographic bone loss (RBLmax), and periodontitis stage. As a secondary exploratory objective, a combined PAD4 and cit-VIM logistic regression model was constructed to evaluate its apparent discriminatory performance for distinguishing periodontitis from periodontal health within the present study sample.

## 2 Materials and methods

### 2.1 Study design and subjects

A single-center cross-sectional case-control study was conducted. Because the study was exploratory, no formal *a priori* sample-size calculation was performed. The sample size was determined by the number of eligible participants with available plasma samples and complete PAD4 and cit-VIM measurements during the study period. A total of 90 participants were included: 30 screened controls with no self-reported history or clinically apparent signs of periodontal disease (HC) and 60 patients with periodontitis (PD). The unequal group sizes resulted from the number of eligible participants available during the recruitment period. Medical histories and general oral examination findings were recorded for all participants. The medical histories included information on systemic diseases and regular medication use. At enrollment, none of the participants had a documented systemic disease or reported regular medication use. Periodontal examinations were performed in the PD group as part of routine clinical care. Given the exploratory design, the findings require confirmation in larger, independently recruited cohorts undergoing standardized periodontal examinations. The study protocol was approved by the Medical Ethics Review Committee of the Stomatological Hospital Affiliated to Anhui Medical University (approval no. T2024002).

### 2.2 Inclusion and exclusion criteria

Participants in the HC group had no self-reported history of periodontitis or current oral symptoms and showed no clinically apparent periodontal abnormalities during general oral screening. They were therefore enrolled as healthy controls. Comprehensive periodontal charting and radiographic assessment were not included in the HC study protocol, and quantitative periodontal indices were consequently unavailable for this group. Radiographs were not routinely obtained from healthy participants for research purposes. Although the participants showed no clinically apparent periodontal abnormalities during general oral screening, individual quantitative PLI, CI, and probing-depth records were not retained in the study database and were therefore not treated as zero. Patients in the PD group were diagnosed and staged in accordance with the 2017 case definition and staging system for periodontitis (Papapanou et al., 2018; Tonetti et al., 2018).

Exclusion criteria were as follows: antibiotic use or comprehensive periodontal treatment within the preceding 3 months; pregnancy or lactation; active infection, active autoimmune disease, malignant tumor, severe hepatic or renal dysfunction; and long-term use of glucocorticoids or immunosuppressants. All blood samples were collected before the initiation of any periodontal treatment, including scaling and root planing.

### 2.3 Periodontal clinical examination and radiographic measurement

Periodontal examinations were performed by experienced periodontal specialists as part of routine clinical care. The study protocol did not specify a minimum number of remaining teeth or the exclusion of third molars. Probing depth was evaluated at clinically examined sites, and the PDmax was defined as the greatest value documented for each participant. The PLI was assessed according to the Silness–Löe Plaque Index on six index teeth (16, 12, 24, 32, 36, and 44). Plaque accumulation at the gingival margin was scored from 0 to 3: 0, no plaque; 1, plaque not visible to the naked eye but detectable by passing a probe across the tooth surface at the gingival margin; 2, moderate visible plaque accumulation at the gingival margin; and 3, abundant plaque within the gingival sulcus and/or at the gingival margin. The participant-level PLI was calculated as the mean score of the examined sites (Silness and Loe, 1964). Calculus was assessed using the Calculus Index–Simplified (CI-S), a component of the Simplified Oral Hygiene Index. Six index surfaces were examined: the facial surfaces of teeth 16, 11, 26, and 31 and the lingual surfaces of teeth 36 and 46. Each surface was scored from 0 to 3: 0, no calculus; 1, supragingival calculus covering no more than one-third of the exposed tooth surface; 2, supragingival calculus covering more than one-third but no more than two-thirds of the surface and/or isolated flecks of subgingival calculus; and 3, supragingival calculus covering more than two-thirds of the surface and/or a continuous heavy band of subgingival calculus. The participant-level CI-S was calculated as the mean score of the examined surfaces (Greene and Vermillion, 1964). Tooth mobility was assessed clinically by applying alternating pressure to the tooth with rigid dental instruments and observing the displacement and direction of movement. Mobility was graded as follows: grade I, buccolingual mobility of less than 1 mm; grade II, mobility of 1–2 mm with both buccolingual and mesiodistal movement; and grade III, mobility exceeding 2 mm with buccolingual, mesiodistal, and vertical movement. The highest mobility grade observed in each participant was recorded as maximum tooth mobility. Periodontitis staging was determined according to the 2017 World Workshop classification, incorporating clinical attachment loss, the severity and extent of alveolar bone loss, the number of teeth lost due to periodontitis, and stage-specific complexity factors.

Panoramic radiographs were used as the primary imaging modality for quantitative measurement of relative bone loss (RBL). When available, periapical radiographs and computed tomography (CT) images were used as supplementary imaging modalities to identify the tooth and interproximal site exhibiting the greatest RBL and to clarify anatomical landmarks that were indistinct on panoramic radiographs. Quantitative RBL measurements were subsequently performed on panoramic radiographs using RadiAnt DICOM Viewer. At the mesial and distal surfaces of each tooth, the distances from the cementoenamel junction (CEJ) to the alveolar bone crest and from the CEJ to the root apex were measured. RBL was calculated as follows:

RBL (%) = (CEJ-to-alveolar crest distance/CEJ-to-root apex distance) × 100% (Schei et al., 1959; Tonetti et al., 2018). For each tooth, the greater of the mesial and distal RBL values was recorded. The highest tooth-level value for each participant was defined as RBLmax and used in subsequent analyses (**Figure 1**).

**Figure 1:**
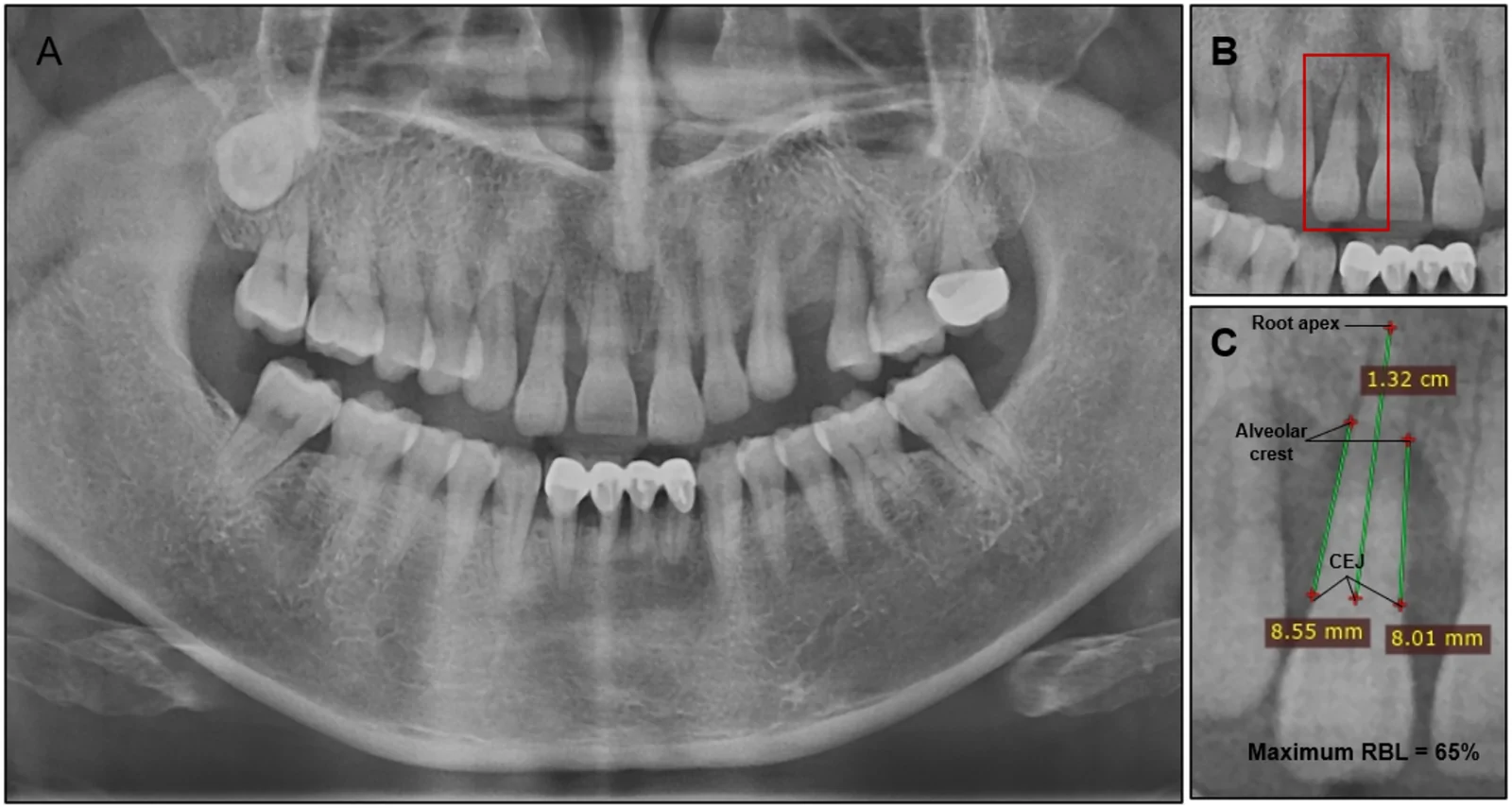
Representative panoramic radiograph and measurement of maximum relative radiographic bone loss. **(A)** Panoramic radiograph of a representative patient with periodontitis, **(B)** Selected tooth and the corresponding region of interest, **(C)** Measurement of radiographic bone loss (RBL). RBL was calculated as the distance from the CEJ to the alveolar crest divided by the distance from the CEJ to the root apex and multiplied by 100%. The greater value measured at the mesial and distal sites was recorded as the maximum RBL. The maximum RBL of the representative tooth was approximately 65%.

Because periodontal information was extracted from clinical records completed by different examiners, some clinical variables were not documented uniformly. RBLmax and periodontitis stage were available only for participants with radiographs of sufficient quality. Analyses were therefore performed using available paired observations without imputation.

### 2.4 Plasma collection and detection of PAD4 and cit-VIM

Fasting peripheral venous blood (2 mL) was collected from each subject into heparin-anticoagulated blood collection tubes and gently mixed by inverting 8–10 times. Within 2 hours after collection, samples were centrifuged at 2,000 × g for 10 min at 4 °C. The supernatant plasma was aliquoted and stored at −80 °C. Samples were slowly thawed on ice prior to detection, and repeated freeze-thaw cycles were avoided (Goodman et al., 2021; Tuck et al., 2009). Plasma PAD4 and cit-VIM concentrations were measured using commercially available ELISA kits according to the manufacturers’ instructions. The human PAD4 ELISA kit was obtained from Nanjing BYabscience Technology Co., Ltd. (Nanjing, China; catalogue no. BY-EH114472). Its analytical range was 0.312–10 ng/mL, with a manufacturer-reported analytical sensitivity of <0.1 ng/mL, intra-assay coefficient of variation (CV) of <10%, inter-assay CV of <15%, and recovery of 85%–115%. The human cit-VIM ELISA kit was obtained from Ruixin Biotechnology (China; catalogue no. RX2D1012026). Its analytical range was 0.25–8 ng/mL, with a manufacturer-reported analytical sensitivity of <0.1 ng/mL, intra-assay CV of <10%, and inter-assay CV of <15%.

Before measurement, each plasma sample was diluted 1:2 with the assay diluent, corresponding to a dilution factor of 2. Each diluted sample was measured in triplicate wells, and the mean of the three measurements was calculated. All measured concentrations fell within the corresponding analytical ranges. The concentration obtained from the standard curve was multiplied by the dilution factor of 2 to determine the final plasma concentration, which was reported in ng/mL. Laboratory personnel were not blinded to clinical group allocation.

### 2.5 Statistical analysis

Statistical analyses and graph plotting were carried out using GraphPad Prism 10.1.2. The normality of continuous variables was evaluated using the Shapiro–Wilk test. Normally distributed continuous variables are presented as mean ± standard deviation, non-normally distributed variables as median and interquartile range, and categorical variables as number and percentage. Between-group comparisons were performed using Welch’s t test or the Mann–Whitney U test for continuous variables and Fisher’s exact test for categorical variables, as appropriate. Associations between plasma biomarkers and periodontal variables were evaluated using Spearman’s rank correlation coefficients. Because periodontitis stage is ordinal, it was also analyzed using Spearman’s rank correlation. Spearman’s correlation coefficients (r_s_), 95% confidence intervals obtained from 5,000 percentile bootstrap resamples, and P values were reported. Benjamini–Hochberg correction was applied across the 12 biomarker–clinical-variable tests, and q<0.05 was considered statistically significant (Benjamini and Hochberg, 1995). Missing clinical measurements were not imputed; each association was analyzed using all available paired observations. Binary logistic regression was performed with periodontal status as the dependent variable, coded as healthy control=0 and periodontitis=1. PAD4 and cit-VIM were entered as continuous variables per 1-ng/mL increase. A biomarker-only model was first fitted, followed by a model adjusted for age, sex, and current smoking. The adjusted logistic regression model included 90 participants, comprising 60 periodontitis events and 30 controls, with five predictor terms: PAD4, cit-VIM, age, sex, and current smoking. Linearity of the continuous predictors on the logit scale was examined using the Box–Tidwell procedure. Receiver operating characteristic (ROC) curves were generated for PAD4, cit-VIM, and the predicted probability from the biomarker-only combined model. Areas under the ROC curves (AUCs) are reported with 95% confidence intervals estimated using DeLong’s method. Correlated AUCs were compared using paired DeLong tests (DeLong et al., 1988). Exploratory cut-offs were selected by maximizing the Youden index, with corresponding sensitivity and specificity (Youden, 1950). The combined model underwent internal validation using 2,000 bootstrap resamples, and an optimism-corrected AUC was calculated (Steyerberg et al., 2001). Analyses were performed using GraphPad Prism 10.1.2 and IBM SPSS Statistics 27.0.1.

## 3 Results

### 3.1 Baseline characteristics of study subjects

A total of 90 subjects were enrolled in this study, including 30 subjects in HC group and 60 subjects in PD group. Baseline characteristics, including age, sex, and current smoking status, are presented in **Table 1**. Participants with periodontitis were older than healthy controls (41.27 ± 11.55 versus 34.50 ± 6.27 years, P<0.001), whereas sex distribution and current smoking status did not differ significantly between the groups. Plasma PAD4 and cit-VIM levels were significantly higher in the PD group than in the HC group. Complete periodontal clinical records were available for PDmax, PLI, and CI in 43 patients and for maximum tooth mobility in 44 patients. RBLmax and periodontitis stage were available for 55 patients with suitable radiographic records. Analyses were performed using available cases without imputation.

**Table 1:** Characteristics and biomarker concentrations of study participants.

Characteristic	Healthy control (n=30)	Periodontitis (n=60)	P value
Sexr			0.823
Male, n (%)	17 (56.7)	31 (51.7)	
Female, n (%)	13 (43.3)	29 (48.3)	
Age, years	34.50 ± 6.27	41.27 ± 11.55	<0.001
Current smoker, n (%)	12 (40.0)	18 (30.0)	0.354
Diabetes, n (%)	0 (0.0)	0 (0.0)	—
Maximum PD, mm (n=43)	—	7.98 ± 2.45	—
Plaque index (n=43)	—	2.00 (1.00–2.00)	—
Calculus index (n=43)	—	1.00 (1.00–2.00)	—
Maximum RBL, % (n=55)	—	36.0 (31.0–44.0)	—
Stage I/II/III/IV, n (n=55)	—	1/15/25/14	—
PAD4, ng/mL	2.456 (2.150–3.099)	3.951 (2.804–5.507)	<0.001
cit-VIM, ng/mL	1.513 (1.163–2.170)	3.559 (2.716–4.593)	<0.001

Data are presented as n (%), mean ± SD, or median (IQR), as appropriate. P values compare HC and PD using Welch’s t test, Fisher’s exact test, or Mann-Whitney U test. Quantitative periodontal indices were not retained for HC and were not assumed to be zero. Available-case denominators are shown for incompletely recorded PD variables. PD, probing depth; RBL, radiographic bone loss.

### 3.2 Plasma cit-VIM and PAD4 concentrations and their association

The median plasma cit-VIM level was 1.513 ng/mL (interquartile range: 1.163, 2.170 ng/mL) in the HC group and 3.559 ng/mL (interquartile range: 2.716, 4.593 ng/mL) in the PD group; plasma cit-VIM concentrations were significantly higher in the PD group than in the HC group (P < 0.0001, **Figure 2A**). The median plasma PAD4 level was 2.456 ng/mL (interquartile range: 2.150, 3.099 ng/mL) in the HC group and 3.951 ng/mL (interquartile range: 2.804, 5.507 ng/mL) in the PD group; plasma PAD4 concentrations were also significantly higher in the PD group than in the HC group (P < 0.0001, **Figure 2B**). Among patients with periodontitis, plasma PAD4 and cit-VIM concentrations showed a moderate positive rank correlation (r_s_=0.459, P<0.001; **Figure 2C**).

**Figure 2:**
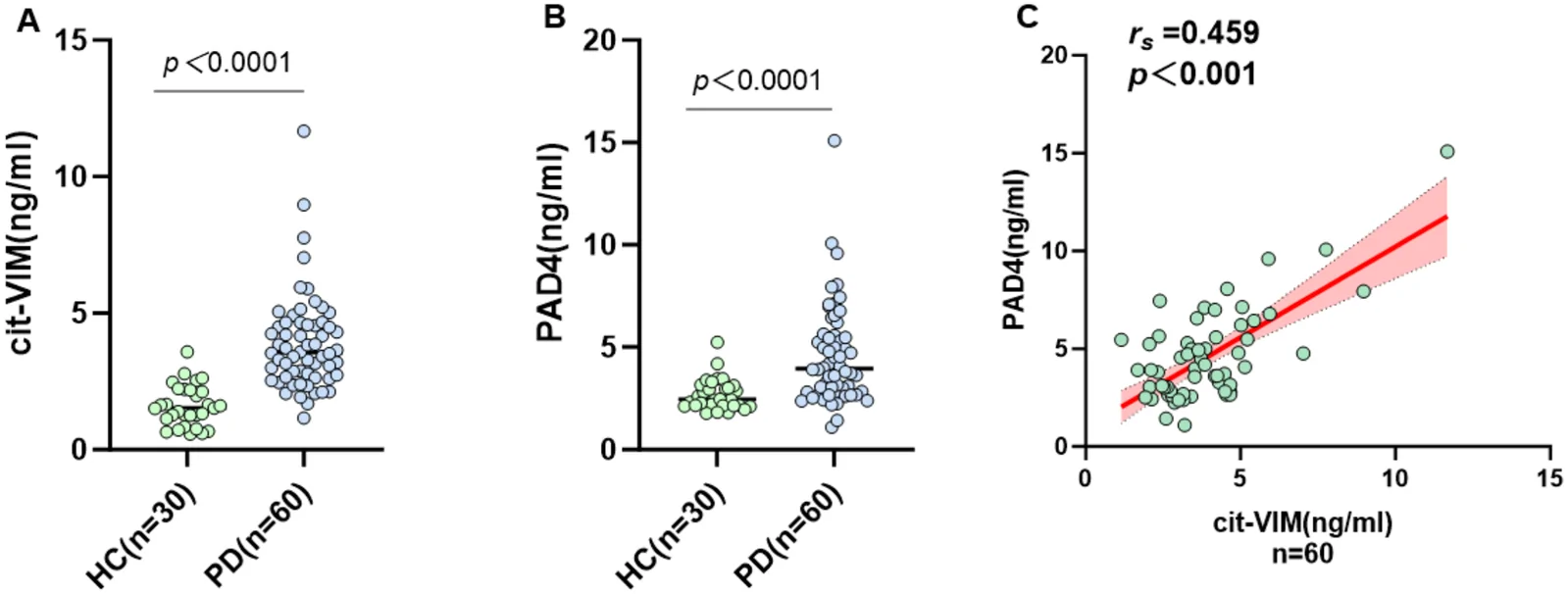
Plasma citrullinated vimentin and PAD4 levels in participants with periodontitis and healthy controls. Plasma levels of **(A)** cit-VIM and **(B)** PAD4 in healthy controls (HC, n=30) and patients with periodontitis (PD, n=60). Horizontal lines and error bars represent the median and interquartile range. Between-group differences were assessed using the Mann–Whitney U test. **(C)** Association between plasma cit-VIM and PAD4 levels among patients with periodontitis (n=60), assessed using Spearman rank correlation analysis. The red line and shaded area indicate the fitted trend line and its 95% confidence interval, respectively. r_s_, Spearman’s rank correlation coefficient.

### 3.3 Correlations between cit-VIM and clinical severity indicators of periodontitis

Plasma cit-VIM was positively associated with PDmax (n=43, r_s_=0.355, 95% CI: 0.043–0.619, q=0.036) and PLI (n=43, r_s_=0.378, 95% CI: 0.079–0.622, q=0.030) after correction for multiple comparisons. Its associations with CI (q=0.097), RBLmax (q=0.097), and periodontitis stage (q=0.097) did not remain statistically significant after correction. No association was observed with maximum tooth mobility (q=0.710; **Figure 3** and **Table 2**).

**Figure 3:**
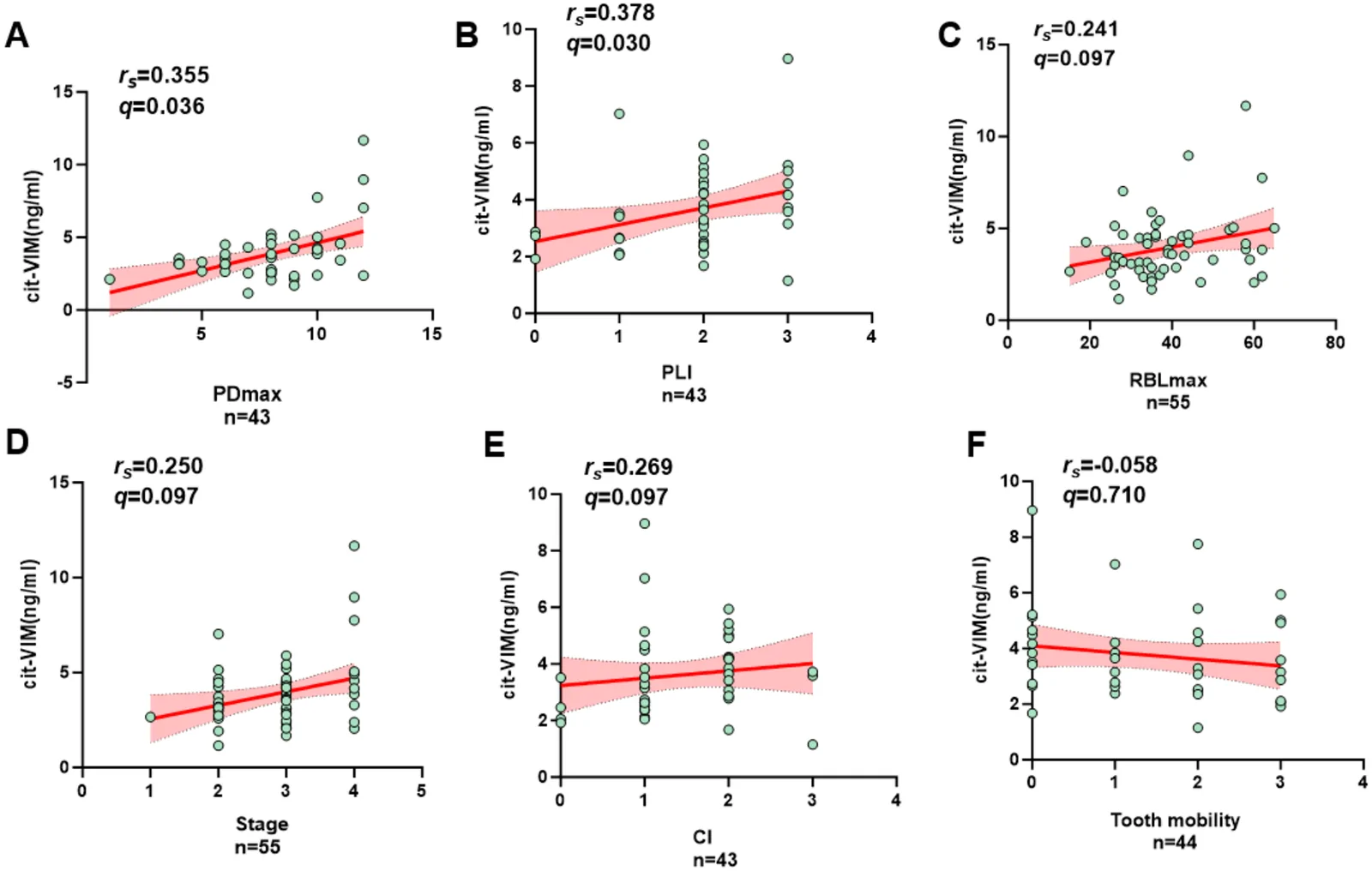
Associations between plasma citrullinated vimentin levels and periodontal clinical parameters. Spearman rank correlation analyses were performed to assess the associations of plasma cit-VIM concentrations with **(A)** PDmax, **(B)** PLI, **(C)** RBLmax, **(D)** periodontitis stage, **(E)** calculus index (CI), and **(F)** maximum tooth mobility. Each circle represents one participant. Red lines and shaded areas indicate the fitted trend lines and corresponding 95% confidence intervals, respectively. Spearman’s correlation coefficients (r_s_) and Benjamini–Hochberg-adjusted q values are shown in each panel. Analyses were performed using available cases without imputation.

**Table 2:** Spearman associations between plasma biomarkers and periodontal clinical variables.

Marker	Variable	n	Spearman ρ	95% CI	Raw P	BH q
cit-VIM	PDmax	43	0.355	0.043–0.619	0.019	0.036
cit-VIM	PLI	43	0.378	0.079–0.622	0.012	0.030
cit-VIM	CI	43	0.269	-0.052–0.556	0.081	0.097
cit-VIM	Mobility	44	-0.058	-0.360–0.250	0.710	0.710
cit-VIM	RBLmax	55	0.241	-0.027–0.485	0.076	0.097
cit-VIM	Stage	55	0.250	-0.028–0.489	0.065	0.097
PAD4	PDmax	43	0.563	0.316–0.743	<0.001	<0.001
PAD4	PLI	43	0.528	0.266–0.723	<0.001	<0.001
PAD4	CI	43	0.350	0.083–0.588	0.021	0.036
PAD4	Mobility	44	0.077	-0.220–0.373	0.619	0.676
PAD4	RBLmax	55	0.553	0.334–0.709	<0.001	<0.001
PAD4	Stage	55	0.626	0.433–0.763	<0.001	<0.001

Confidence intervals were obtained from 5,000 percentile bootstrap resamples. Benjamini-Hochberg q values control the false discovery rate across the 12 marker-clinical tests. Analyses used available paired observations without imputation.

### 3.4 Associations between plasma PAD4 and periodontal clinical and radiographic parameters

Plasma PAD4 was positively associated with PDmax (n=43, r_s_=0.563, q<0.001), PLI (n=43, r_s_=0.528, q<0.001), CI (n=43, r_s_=0.350, q=0.036), RBLmax (n=55, r_s_=0.553, q<0.001), and periodontitis stage (n=55, r_s_=0.626, q<0.001). No significant association was observed between PAD4 and maximum tooth mobility (n=44, r_s_=0.077, q=0.676; **Figure 4** and **Table 2**).

**Figure 4:**
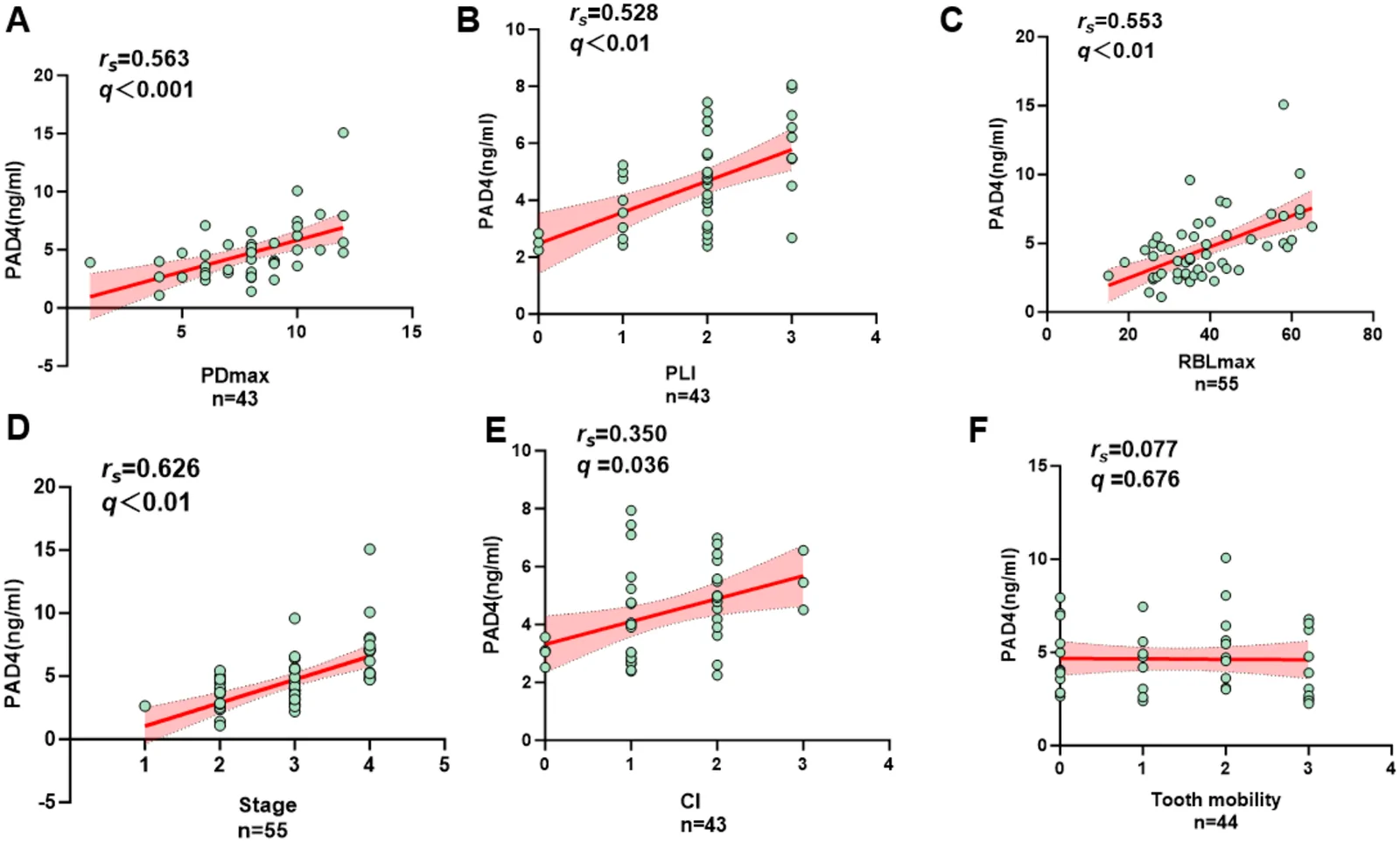
Associations between plasma PAD4 levels and periodontal clinical parameters. Spearman rank correlation analyses were performed to assess the associations of plasma PAD4 concentrations with **(A)** PDmax, **(B)** PLI, **(C)** RBLmax, **(D)** periodontitis stage, **(E)** calculus index (CI), and **(F)** maximum tooth mobility. Each circle represents one participant. Red lines and shaded areas indicate the fitted trend lines and corresponding 95% confidence intervals, respectively. Spearman’s correlation coefficients (r_s_) and Benjamini–Hochberg-adjusted q values are shown in each panel. Analyses were performed using available cases without imputation.

### 3.5 Logistic regression analysis of periodontal status

In the biomarker-only model, PAD4 (OR = 2.84 per 1-ng/mL increase, 95% CI: 1.25–6.45, P = 0.013) and cit-VIM (OR = 12.94, 95% CI: 3.71–45.15, P<0.001) were associated with periodontitis. After adjustment for age, sex, and current smoking, PAD4 (OR = 2.90, 95% CI: 1.21–6.96, P = 0.017) and cit-VIM (OR = 12.70, 95% CI: 3.43–46.96, P<0.001) remained statistically significant. Age, sex, and current smoking were not statistically significant in the adjusted model (**Table 3**). The Box–Tidwell analysis showed no significant departure from linearity in the logit for PAD4 or cit-VIM in the biomarker-only model (interaction P = 0.196 and P = 0.214, respectively; joint likelihood-ratio P = 0.263). In the covariate-adjusted model, the interaction terms for PAD4, cit-VIM, and age were also not statistically significant (P = 0.150, P = 0.099, and P = 0.501, respectively; joint likelihood-ratio P = 0.273).

**Table 3:** Binary logistic regression models for periodontitis status.

Model	Term	β	OR	95% CI	P value
Combined biomarker-only	Intercept	-8.695	0.00	0.00–0.01	<0.001
Combined biomarker-only	PAD4 (per 1 ng/mL)	1.043	2.84	1.25–6.45	0.013
Combined biomarker-only	cit-VIM (per 1 ng/mL)	2.561	12.94	3.71–45.15	<0.001
Combined adjusted	Intercept	-9.853	0.00	0.00–0.03	0.003
Combined adjusted	PAD4 (per 1 ng/mL)	1.064	2.90	1.21–6.96	0.017
Combined adjusted	cit-VIM (per 1 ng/mL)	2.542	12.70	3.43–46.96	<0.001
Combined adjusted	Age (per year)	0.043	1.04	0.94–1.16	0.434
Combined adjusted	Male sex	-0.199	0.82	0.11–6.10	0.846
Combined adjusted	Current smoking	-0.889	0.41	0.04–4.81	0.479

Outcome coding was PD = 1 and HC = 0. Biomarkers were entered continuously per 1 ng/mL. The adjusted model includes age (per year), male sex, and current smoking. OR, odds ratio; CI, confidence interval.

### 3.6 ROC analysis of PAD4, cit-VIM and their combined model

ROC curve analysis revealed that the AUC of PAD4 for discriminating PD from HC was 0.8033 (**Figure 5A**), and the AUC of cit-VIM was 0.9311 (**Figure 5B**). The AUC was 0.803 (95% CI: 0.712–0.894) for PAD4, 0.931 (95% CI: 0.880–0.982) for cit-VIM, and 0.946 (95% CI: 0.901–0.990) for the biomarker-only combined model. At the exploratory Youden-index-derived cut-offs, PAD4 showed a sensitivity of 61.7% and specificity of 93.3%, cit-VIM showed a sensitivity of 78.3% and specificity of 93.3%, and the combined model showed a sensitivity of 96.7% and specificity of 80.0%. Paired DeLong tests showed that the combined model had a significantly higher AUC than PAD4 alone (ΔAUC=0.142, P = 0.001), whereas its AUC was not significantly different from that of cit-VIM alone (ΔAUC=0.014, P = 0.385). The combined model showed exploratory discriminatory performance within the present sample. Because the model was developed and evaluated in the same cohort, these results represent internally estimated performance and do not establish clinical diagnostic utility. The apparent AUC of the combined model was 0.946, and the 2,000-resample bootstrap optimism-corrected AUC was 0.941. These estimates represent internal performance within the present sample and do not constitute external validation. The detailed exploratory discrimination results, including AUCs, Youden-index-derived cut-offs, sensitivity, specificity, and internal validation results, are summarized in **Table 4**.

**Figure 5:**
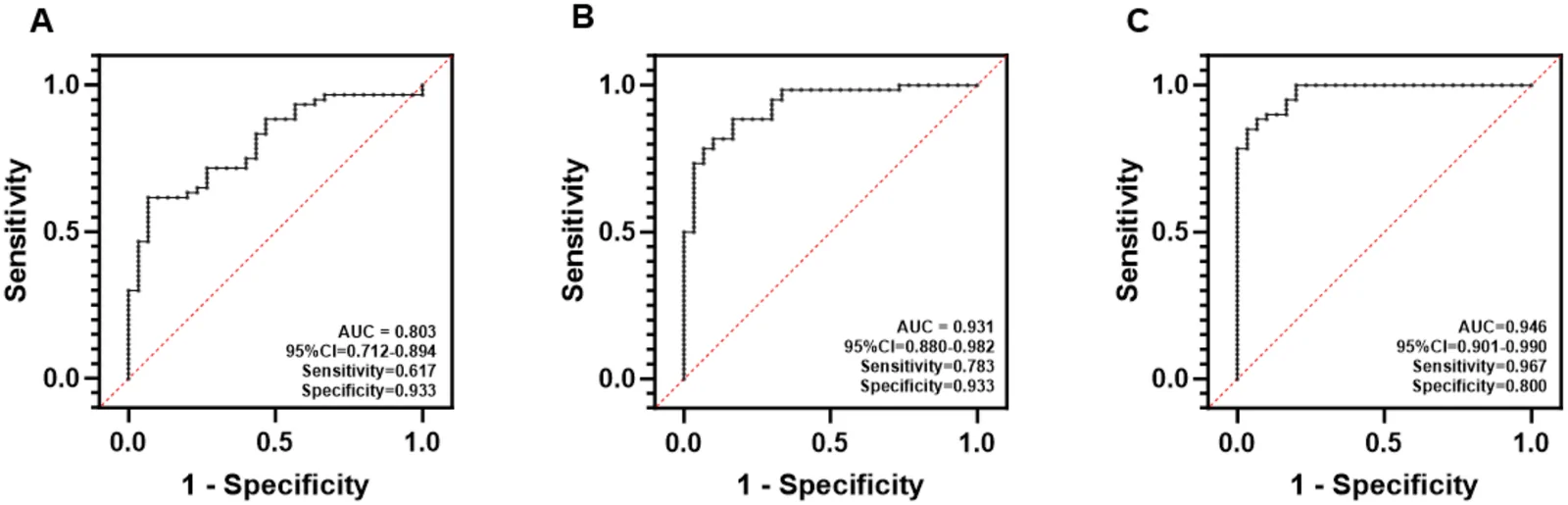
Receiver operating characteristic curves for plasma PAD4, citrullinated vimentin, and their combined model in discriminating periodontitis from periodontal health. ROC curves are presented for **(A)** plasma PAD4, **(B)** plasma cit-VIM, and **(C)** the biomarker-only logistic regression model combining PAD4 and cit-VIM. The areas under the curve (AUCs) were 0.803 (95% CI: 0.712–0.894), 0.931 (95% CI: 0.880–0.982), and 0.946 (95% CI: 0.901–0.990), respectively. Sensitivity and specificity were calculated at the exploratory cut-offs determined using the maximum Youden index. The red diagonal line represents discrimination expected by chance. CI, confidence interval; ROC, receiver operating characteristic.

**Table 4:** Exploratory discrimination and internal validation.

Marker/model	AUC	95% CI	Youden cut-off	Sensitivity	Specificity
PAD4	0.803	0.712–0.894	3.567 ng/mL	61.7%	93.3%
cit-VIM	0.931	0.880–0.982	2.628 ng/mL	78.3%	93.3%
PAD4 + cit-VIM	0.946	0.901–0.990	0.360 probability	96.7%	80.0%

AUC confidence intervals used DeLong’s method. Paired DeLong comparisons: combined versus PAD4, difference=0.142, P = 0.001; combined versus cit-VIM, difference=0.014, P = 0.385. The 2,000-resample optimism-corrected AUC for the combined model was 0.941. Cut-offs are exploratory and require external validation.

## 4 Discussion

This study investigated the association of plasma PAD4, cit-VIM and periodontitis. The results demonstrated that plasma PAD4 and cit-VIM levels were significantly elevated in patients with periodontitis, and a moderate positive rank correlation was observed between the two biomarkers within the periodontitis group. After correction for multiple comparisons, cit-VIM remained associated with PDmax and PLI, whereas PAD4 remained associated with PDmax, PLI, CI, RBLmax, and periodontitis stage. ROC analysis showed that cit-VIM exhibited superior discriminatory performance compared with PAD4. The combined model showed higher apparent discrimination than PAD4 alone but did not significantly outperform cit-VIM alone. PAD4 catalyzes the citrullination of histone and non-histone substrates and participates in the formation of NETs (Mondal and Thompson, 2019). Persistent microbial stimulation within periodontal pockets recruits and activates neutrophils, facilitating the release of proteases, histones and citrullinated proteins, which in turn exacerbates local inflammation and tissue destruction (Ando et al., 2024; Hajishengallis, 2020; Wang et al., 2021). At the tissue level, Engström et al. reported increased PAD2 and PAD4 expression and increased global protein citrullination specifically in the gingival connective tissue of patients with periodontitis (Engström et al., 2018). Harvey et al. detected PAD2, PAD4, and citrullinated proteins in gingival tissue and found that their protein expression was associated with the severity of local inflammation (Harvey et al., 2013). Nesse et al. reported increased global citrullinated-protein staining in periodontitis-affected periodontal stroma but did not evaluate PAD expression or cit-VIM specifically (Nesse et al., 2012). In isolated neutrophils, *P. gingivalis* lipopolysaccharide induced NET formation through a Ca²^+^–TPL2–MEK–ERK–PAD4 signaling pathway (Chen et al., 2022). In the present study, plasma PAD4 concentrations were higher in the periodontitis group and positively correlated with PDmax, PLI, RBLmax and Stage, indicating that circulating PAD4 concentrations were associated with clinical and radiographic measures of periodontal disease burden in this sample of patients with periodontitis.

Cit-VIM is generated through PAD-mediated citrullination of vimentin, a cytoskeletal intermediate-filament protein (Mondal and Thompson, 2019). Plasma cit-VIM may reflect one component of citrullination-related inflammatory processes; however, it cannot be assumed to represent total tissue citrullination, overall inflammatory-cell activity, or the tissue source of the modified protein. Inflammatory-cell activation, cell injury, and NET-related processes may increase extracellular vimentin exposure and create conditions that favor its citrullination, although the source and pathway responsible for circulating cit-VIM in periodontitis remain uncertain (Mondal and Thompson, 2019; Suprewicz et al., 2025). *In vitro*, both extracellular native vimentin and citrullinated vimentin modulated human neutrophil functions through TLR4-related signaling, although their effects differed across individual neutrophil responses (Suprewicz et al., 2025). Whether this mechanism operates in periodontitis remains unknown. Evidence from periodontal tissues and gingival crevicular fluid concerns different components of the citrullination process. Increased PAD2/PAD4 expression and global protein citrullination have been reported in gingival connective tissue from patients with periodontitis (Engström et al., 2018), while the tissue expression of PAD2, PAD4, and citrullinated proteins has been associated with the severity of local gingival inflammation (Harvey et al., 2013). Increased global citrullinated proteins, rather than cit-VIM specifically, have also been demonstrated in periodontitis-affected periodontal tissue (Nesse et al., 2012). In gingival crevicular fluid, human PAD and bacterial PPAD activities have been detected, with higher PPAD activity reported in participants with periodontitis (Laugisch et al., 2016). These local findings provide biological plausibility for investigating citrullination-related circulating markers. However, none of these studies demonstrated that plasma cit-VIM originates from periodontal tissues or that local PAD expression directly accounts for the elevated plasma cit-VIM observed in the present study. The positive association between PAD4 and cit-VIM is compatible with coordinated changes in citrullination-related processes. However, it does not demonstrate PAD4 enzymatic activity or direct PAD4-mediated citrullination of vimentin.

In terms of clinical parameters, in the present sample, PAD4 showed numerically larger Spearman correlation coefficients with RBLmax and periodontitis stage than cit-VIM. However, the correlation coefficients were not formally compared, and these numerical differences should not be interpreted as evidence that PAD4 is more strongly associated with these outcomes. RBLmax and stage primarily represent cumulative alveolar bone loss and disease complexity. These cross-sectional associations are biologically compatible with relationships among neutrophil activation, citrullination, and periodontal destruction (Ando et al., 2024; Hajishengallis,2020; Shindo et al., 2023), but their temporal or causal direction cannot be determined. Tooth mobility is influenced by multiple factors, including the distribution of bone loss, occlusal loading, and root anatomy, which may partly explain its weak association with circulating biomarkers.

ROC analysis showed that PAD4 and cit-VIM had apparent discriminatory ability within the present sample, with cit-VIM demonstrating a higher AUC than PAD4. The biomarker-only combined model produced an apparent AUC of 0.946 and an optimism-corrected AUC of 0.941. Although the combined model significantly outperformed PAD4 alone, it did not significantly outperform cit-VIM alone. Therefore, the present results do not establish a clear incremental benefit of combining the two biomarkers. The identified cut-offs are exploratory and should not be interpreted as clinically validated diagnostic thresholds (Collins et al., 2015).

Several limitations should be acknowledged. First, this single-center, cross-sectional study had a modest sample size and therefore cannot establish causal relationships. PAD4 and cit-VIM were measured only in plasma at a single time point, and their circulating levels may not reflect the local periodontal microenvironment. Moreover, PAD4 concentration rather than enzymatic activity was assessed, and NET-related markers were not measured. Laboratory personnel were not blinded to clinical group allocation, although standardized procedures, triplicate measurements, and objective optical-density readings were used. Based on the medical histories recorded at enrollment, none of the included participants had a documented systemic disease or reported current regular medication use. However, detailed information on intermittent or short-term medication exposure, including the specific drug, dosage, and duration, was not systematically collected. In addition, although pregnant and lactating women were excluded, other hormone-related factors among female participants, such as menstrual-cycle phase, menopausal status, and the use of hormonal contraceptives or hormone-replacement therapy, were not systematically recorded. Transient minor illnesses or inflammatory conditions, such as common upper respiratory infections, were also not comprehensively documented. These unmeasured medication-related, hormonal, and inflammatory factors may have influenced circulating PAD4 and cit-VIM concentrations; therefore, residual confounding cannot be completely excluded.

Second, incomplete clinical records resulted in different sample sizes across association analyses and may have introduced selection bias. The limited and uneven distribution of periodontitis stages precluded adequately powered stage-specific analyses, while PDmax did not fully represent disease extent or total inflammatory burden. Panoramic radiographs are susceptible to magnification, geometric distortion, and superimposition (Jacobs et al., 2024), and quantitative periodontal and radiographic data were unavailable for the healthy controls. In addition, extreme biomarker values and the modest sample size contributed to uncertainty in the logistic regression estimates (Riley et al., 2020). Because the model was developed and evaluated in the same cohort and no participants with gingivitis or other inflammatory diseases were included, periodontal specificity and clinical diagnostic utility cannot be established. Bootstrap correction reduces optimism but does not replace independent external validation (Collins et al., 2015; Steyerberg et al., 2001).

Future studies should use larger, multicenter, prospective cohorts with clinically relevant comparison groups. Parallel assessment of biomarkers in plasma, saliva, gingival crevicular fluid, and gingival tissues, together with PAD4 activity and NET-related markers, may clarify their biological sources and mechanisms. Independent validation is also required to confirm the discriminatory performance and exploratory cut-offs of the combined model.

## 5 Conclusions

Plasma PAD4 and cit-VIM concentrations were elevated in patients with periodontitis and were associated with periodontal status. PAD4 was also associated with several clinical and radiographic measures of periodontal disease burden. The combined model demonstrated exploratory discriminatory ability within the present sample but did not significantly outperform cit-VIM alone. These findings require confirmation in larger, clinically diverse, and independent cohorts.

## Data Availability

The raw data supporting the conclusions of this article will be made available by the authors, without undue reservation.
